# “Practice so that the skill does not disappear”: mixed methods evaluation of simulator-based learning for midwives in Uganda

**DOI:** 10.1186/s12960-019-0350-z

**Published:** 2019-03-29

**Authors:** Emma Williams, Eva S. Bazant, Samantha Holcombe, Innocent Atukunda, Rose Immaculate Namugerwa, Kayla Britt, Cherrie Evans

**Affiliations:** 1 Jhpiego, 1615 Thames St, Baltimore, MD 21231 USA; 2 Nakawa Division, Plot 36, Lower Naguru, East Road, Kampala, Uganda; 3 Save the Children, Plot 2163, Dadiri Close, Muyenga, P.O. Box 12018, Kampala, Uganda

## Abstract

**Background:**

Postpartum hemorrhage and neonatal asphyxia are leading causes of maternal and neonatal mortality, respectively, that occur relatively rarely in low-volume health facilities in sub-Saharan Africa. Rare occurrence of cases may limit the readiness and skills that individual birth attendants have to address complications. Evidence suggests that simulator-based training and practice sessions can help birth attendants maintain these life-saving skills; one approach is called “low-dose, high-frequency” (LDHF). The objective of this evaluation is to determine the facilitating factors and barriers to participation in LDHF practice, using qualitative and quantitative information.

**Methods:**

A trial in 125 facilities in Uganda compared three strategies of support for LDHF practice to improve retention of skills in prevention and treatment of postpartum hemorrhage and neonatal asphyxia. Birth attendants kept written logs of their simulator-based practice sessions, which were entered into a database, then analyzed using Stata to compare frequency of practice by the study arm. The evaluation also included 29 in-depth interviews and 19 focus group discussions with birth attendants and district trainers. Transcripts were entered in Atlas.ti software for coding, then analyzed using content analysis to identify factors that motivated or discouraged simulator-based practice.

**Results:**

Practice log data indicated that simulator-based practice sessions occurred more frequently in facilities where one or two practice coordinators helped schedule and lead the practice sessions and in health centers compared to hospitals. The qualitative data suggest that birth attendants who practiced more were motivated by a desire to maintain skills and be prepared for emergencies, external recognition, and establishing a set schedule. Barriers to consistent practice included low staffing levels, heavy workloads, and a sense that competency can be maintained through routine clinical care alone. Some facilities described norms around continuing education and some did not.

**Conclusions:**

Designating practice coordinators to lead their peers in simulator-based practice led to more consistent skills practice within frontline health facilities. Ongoing support, scheduling of practice sessions, and assessment and communication of motivation factors may help sustain LDHF practice and similar forms of continuing professional development.

**Trial registration:**

Registered with clinicaltrials.gov #NCT03254628 on August 18, 2018 (registered retrospectively).

**Electronic supplementary material:**

The online version of this article (10.1186/s12960-019-0350-z) contains supplementary material, which is available to authorized users.

## Background

Many sub-Saharan African countries have high maternal mortality ratios and neonatal mortality rates, even though most births occur in health facilities. For example, Uganda has maternal mortality ratio of 336 deaths per 100 000 live births and a neonatal mortality rate of 27 per 1000 live births, coupled with a 73% institutional delivery rate [1]. This suggests that improvements in quality of obstetric care are needed to reduce maternal and newborn mortality in Uganda and similar settings [2–5]. Improving quality requires ensuring that birth attendants have the skills and equipment necessary to provide essential care. Traditional capacity-building models for health care providers in low- and middle-income countries have involved off-site sessions that may last 1 week or longer, often with limited follow-up afterwards. However, these approaches may not lead to sustained improvements in clinical practices and outcomes. Within the past decade, synthesis of the research evidence has suggested that short team learning sessions at the health facility employing simulation, followed by simulation-based practice, can improve learning outcomes and provider performance compared with training approaches that do not include subsequent practice [6–8]. This approach of short onsite training sessions combined with simulation-based practice is referred to here as “low-dose, high-frequency” (LDHF). Evidence suggests that simulation-based training sessions can improve skills and clinical outcomes [6, 9]. Simulation is accepted as an effective strategy for nursing and medical education to supplement didactic education and required in-service clinical practice hours in the United States of America and other high-income countries [10]. Use of simulation is being introduced more frequently in low- and middle-income countries [11]. The World Health Organization (WHO) standards for improving maternal and newborn care in facilities recommend monthly drills or simulation exercises to maintain skills around complication readiness [2]. However, little research has examined how to effectively scale up simulation-based practice within health systems in low-resource settings.

Two key technical areas that frequently require skills strengthening for improved quality of care are prevention and treatment of postpartum hemorrhage, the leading cause of maternal death in sub-Saharan Africa, and neonatal resuscitation to address birth asphyxia, a largely preventable cause of newborn death. Since postpartum hemorrhage (PPH) and neonatal asphyxia occur relatively infrequently in any given facility, particular lower volume facilities at lower levels of the health system, refresher training sessions can be used to help birth attendants maintain their skills, knowledge, and confidence. Two examples of refresher sessions are Helping Babies Breathe™ (HBB) and Helping Mothers Survive Bleeding after Birth™ (HMS BAB) both of which utilize the LDHF approach. HBB is a one-day basic newborn resuscitation course using the NeoNatalie™ simulator. HBB is intended to be followed by brief, frequent practice sessions using the simulator for skills retention. In Ghana, India, Kenya, and Tanzania, published findings have shown that this approach is associated with improved clinical competencies and perinatal mortality [12–16]. HMS BAB is a single-day training course on postpartum hemorrhage prevention and treatment using the MamaNatalie simulator. Similarly, HMS BAB is designed to be followed with brief practice sessions. HMS BAB evaluations found immediate improvement in knowledge, skills and confidence, maintenance of confidence and simulated skills over time [17], and improvements in clinical outcomes following the training course [18]. However, few published reports have described the details of how to implement HBB, HMS BAB, or other LDHF training approaches, such as frequency of practice sessions and how to motivate practice, particularly outside the context of clinical trials in controlled settings [19]. These aspects of implementation are essential to determine how LDHF approaches can be integrated into standard in-service capacity building.

In the parent study to this study, a pragmatic, cluster-randomized trial design [20] was used to evaluate three modalities of peer-assisted learning after HMS BAB and HBB training sessions. The trial found improvements in clinical care, including uterotonic provision within the first minute after birth, and a decline in intrapartum stillbirth, neonatal mortality, PPH, and retained placenta from baseline to endline in all study arms [21]. This article describes a nested mixed methods evaluation focusing on practice using simulators. The two objectives are (a) to describe factors contributing to birth attendants’ participation in LDHF practice, as identified by birth attendants and managers, and (b) to explore differences among hospitals and health centers in factors that motivated or discouraged LDHF practice.

## Methods

**Fig. 1:**
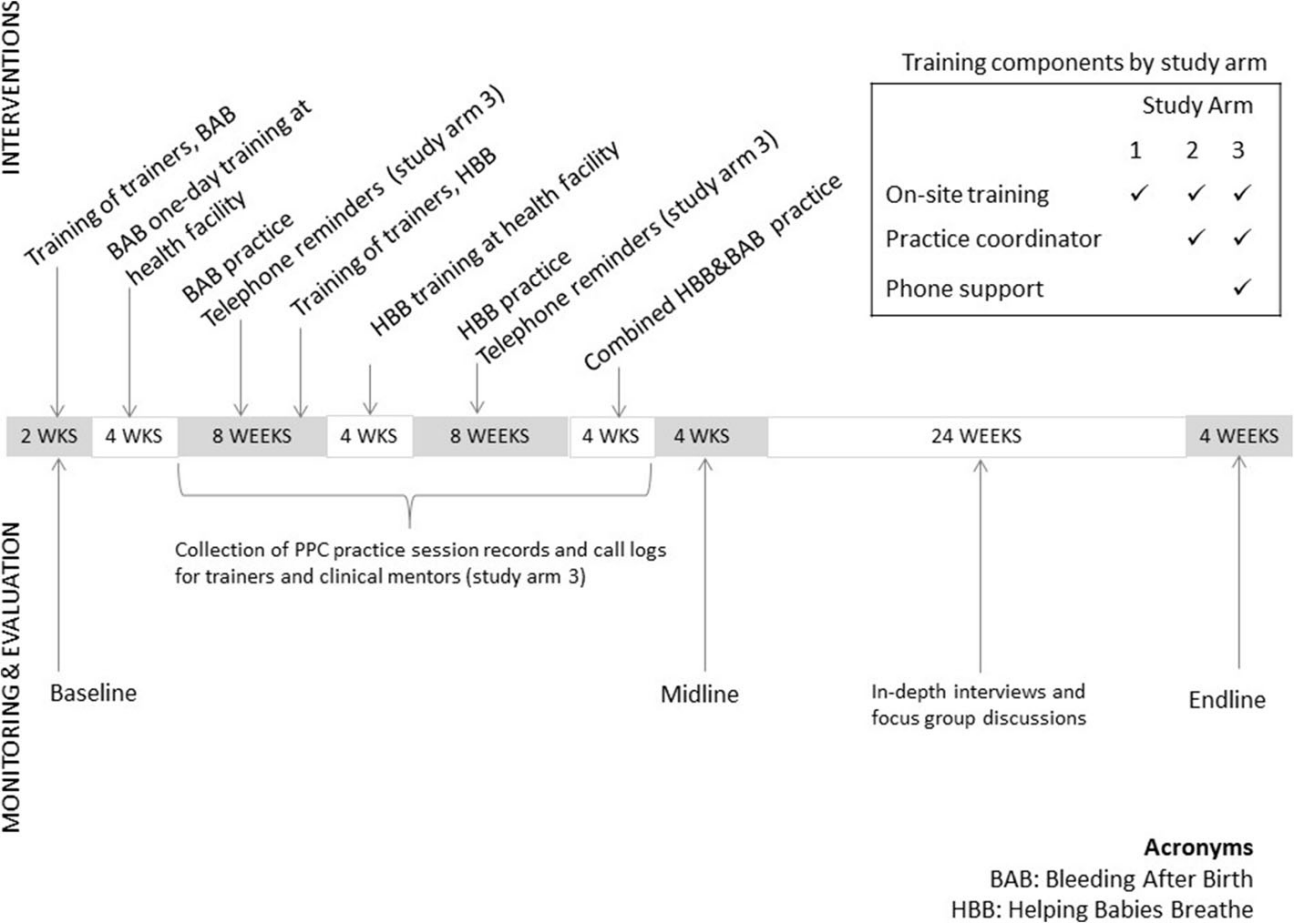
Timeline of study intervention components and data collection presented in this manuscript

The study data presented here represent a sequential explanatory design [22], with the qualitative data collection following the quantitative data collection to help interpret the quantitative findings (Fig. 1). Records of providers’ participation in LDHF practice sessions were analyzed descriptively to understand fidelity to the intervention across study arms and facility types.

### Description of intervention and main study design

First, with regard to the main study described in detail elsewhere [21], the study took place in 12 districts at 125 health facilities in the Western and Eastern regions of Uganda in 2014–2016. All public maternities in selected districts participated, including health centers III, which had lower monthly birth volume and were staffed by a few midwives, and health centers IV and hospitals, which had higher volume and staffing.

All facilities in all study arms received brief onsite training sessions. Ugandan midwives certified as trainers led the 1-day HMS BAB training session at each facility, and all maternity unit staff were invited to participate, not just doctors, midwives, and nurses. The session included the expectation that maternity unit staff practice on a simulator for 10–15 min once per week for the following 8 weeks. After the 2-month practice period ended, the same trainers led the 1-day HBB training session and again suggested practice with specific scenarios for 10–15 min once per week for 8 weeks. For an additional 4 weeks, maternity unit staff were asked to practice scenarios including care for both mother and newborn.

In study arms 2 and 3, practice sessions were facilitated by one of the birth attendants at the facility designated as the peer practice coordinator who received an extra half-day orientation, while in study arm 1, no one was assigned to coordinating or leading practice sessions (Fig. 1). Local stakeholders preferred to use the term “clinical mentor.” For clarity, we are referring to the clinical mentors as “practice coordinators” in this paper. Practice coordinators received no extra compensation but were reimbursed for travel costs to deliver program monitoring and evaluation records. In study arm 3 only, district trainers were asked to call peer practice coordinators to encourage practice sessions, and mobile phone airtime credits were provided. The study team provided detailed practice session plans to all facilities’ coordinators and health care workers.^1^ These sessions were recommended to occur during quiet periods on the labor ward or at the beginning or end of a provider’s shift. Practice could happen in small groups or individually with the practice coordinators. Many of the practice sessions provided opportunities for all maternity unit staff to practice together, not just birth attendants. This included recognizing warning signs, using clear communication, maintaining stock and correctly storing materials, and retrieving drugs and supply materials for birth attendants if needed.

### Data collection and entry of structured tools

Three structured study tools were used to collect quantitative data. First, study staff listed all maternity unit staff who “perform or assist” deliveries at the facility, including names and cadres, using a paper form. This list was updated before each training session and at two subsequent data collection points: after the 6-month period of HMS BAB and HBB and after 20 weeks of practice (called midline). Second, each peer practice coordinator, (in study arms 2 or 3) or another designated person within the facility, (in study arm 1) was given a paper logbook on which to record practice sessions and asked to document practice session details, including date, provider’s name and cadre, topic practiced, and length of session. District trainers collected the HMS practice logs during the HBB training session. After finding low compliance with record-keeping around practice documentation after HMS, project staff reminded practice coordinators (study arms 2 and 3) and designated persons (study arm 1) of the importance of maintaining practice logs. Data collectors retrieved the HBB logs during the midline data collection. Project data entry staff entered the information from paper logs to a Microsoft Access database created with internal consistency checks. Other project staff checked the original paper records against the database. This database was merged with a list of all birth attendants assigned to each facility’s maternity unit, using the provider’s name and ID number, through a combination of the merge functions in Stata version 15 [23] and manual checks by study staff. Third, in study arm 3 only, district trainers were asked to telephone the practice coordinators to encourage them to hold practice sessions and to keep written records of their calls. Call records were entered into a Microsoft Access database and analyzed in Microsoft Excel.

### Data analysis of structured tools

Several indicators were compared across study arms: (1) the proportion of facilities submitting a provider practice log following HMS and HBB and the combined practice period, (2) the mean number of practice sessions and 95% confidence interval per month per provider over 2-month periods (July–August, September–October, and November–December) for each type of training content^2^, (3) the total number of practice sessions per provider (for HMS and HBB combined, over the project duration) was calculated at the facility level, and the mean for all facilities, (4) the mean and 95% confidence interval of total practice sessions per provider stratifying by the following factors: (a) higher (above the total mean) or lower documented practice, (b) higher level of facility (hospital or health center IV) or lower level, (c) study arms with a practice coordinator (study arms 2 and 3), or study arm 1. All quantitative data analysis was completed using Stata version 15.

### Data collection and entry of qualitative data

In May and June 2015, focus group discussions and semi-structured interviews were conducted with nurses, midwives, and health facility in-charges in 24 selected facilities, in order to gather contextual information about the project intervention and rich descriptions of the training and practice sessions. The sample was stratified based on the study arm and level of simulator practice sessions achieved, and a preliminary analysis of whether the facility had achieved higher- or lower-than-average practice levels. Districts and then facilities were purposively sampled, using data from the practice logs and study team members’ knowledge of contextual factors. Each group of 12 high- and 12 low-practice facilities included different levels of health facilities; the hospital from each district was included. The sample size was selected with the aim of achieving saturation in the higher and lower practice subgroup in each study arm.

The study team contacted facilities prior to the day of data collection to schedule the focus group discussions; however, sometimes communication gaps and scheduling challenges—such as providers going back to school, on maternity leave, and being out for illness or on leave—limited data collection. The study team did not track refusals, but refusals were unusual because the study team adjusted their activities to accommodate participants’ schedules. Planned and achieved data collection is presented in Table 1. Within each facility, data collectors invited all staff who assisted with or conducted deliveries to participate in the focus group discussion. They recruited the practice coordinator (or facility in-charge, in control districts) in each selected facility and the district trainer in each district to participate in in-depth interviews. Two Ugandan researchers with graduate-level education in qualitative research conducted the interviews and focus group discussions in English using an interview guide that had been pre-tested in facilities outside the study area. Interview and focus group discussion topics included experiences with the two training sessions, perceived challenges in completing practice sessions and strategies to overcome those challenges, recommendations for improving the LDHF approach, and any perceived changes in clinical practice following the training and practice sessions. Interviews and focus group discussions lasted about 1 h. For the participants’ convenience, data collection took place at or near the health facility where they worked, in a setting that enabled auditory privacy, without any non-participants present. No follow-up interviews or focus group discussions were conducted. The interviewers were not involved in other aspects of study implementation and received a 2-day orientation related to the qualitative component’s objectives, tools, consent process, research ethics, recruitment and interview, and focus group moderation techniques. The interviewers made field notes on the day of data collection and, within a few weeks of the interviews, transcribed their own audio recordings verbatim. Transcripts were spot-checked for quality against the audio recordings by a study co-investigator.

**Table 1:** Sample size for qualitative data collection, by adherence level to practice guidelines

	Facilities with Higher Adherence Total (per study arm)	Facilities with Lower Adherence Total (per study arm)	Total
Planned data collection			
Facilities^1^	12 (4)	12 (4)	24 (8)
Focus group discussions	12 (4)	12 (4)	24 (8)
Interviews with practice coordinators (or proxies)^3^	12 (4)	12 (4)	24 (8)
Interviews with district trainers	3 (1)	3 (1)	6 (2)
Completed data collection			
Facilities	10	10	20
Focus group discussions	3	9	12
Interviews with practice coordinators	6	8	14
Interviews with district trainers^2^			6

^1^Sample was split evenly between Eastern and Western regions. All facilities per study arm were in the same district

^2^All districts sampled included both high and low practice facilities

^3^Practice coordinators in control arm had a proxy for the qualitative study

### Qualitative data analysis

Transcripts of focus group discussions and interviews were coded in Atlas.ti version 7 [24] using a codebook based on predetermined and emergent codes. Four study co-investigators and one interviewer coded the interviews following an exercise of coding an initial sample of interviews to achieve consistency. The analysts communicated frequently about how the codes were being applied and the process of coding transcripts. In the interest of reflexivity, investigators made an effort to be aware of own biases, such as being predisposed to think that LDHF is an effective training approach, and our own limitations as outsiders to the participating health facilities. A qualitative content analysis approach was used to identify themes and answer research questions [25], influenced by Friese’s Noticing-Collecting-Thinking approach [26]. After coding each transcript, Atlas.ti enabled generation of query “reports” of all data from similar codes; these reports were produced separately for facilities in the higher and lower practice categories, and by type of facility (hospitals vs. from health centers) and study group (study arm 2 or 3 vs. 1). Analysts conducted multiple reviews of reports and identified themes present across transcripts and determined which themes were expressed more strongly within participant subgroups.

### Ethics, consent, and permissions

Institutional Reviews Boards at the Johns Hopkins Bloomberg School of Public Health in Baltimore, Maryland, United States, and Makerere University in Kampala, Uganda and the Ugandan government approved this study. All participants provided verbal informed consent prior to participation, as per the approved protocol. The study is registered on clinicaltrials.gov #NCT03254628.

## Results

The quantitative sample included 125 facilities, with data from 785 health care workers during the post-HMS practice sessions and 814 during the post-HBB practice sessions; demographic information was not included in this data collection tool. The qualitative sample included 11 district trainers, 23 practice coordinators (study arms 2 and 3) or 5 facility in-charges (study arm 1), and 68 focus group discussion participants (Table 1); each focus group discussion included an average of five participants. Among all qualitative participants, the mean age was 36 (standard deviation 10), 90% were women, and their cadres were mainly midwife (40%), nurse or nursing officer (27%), or nursing assistant (12%). The factors that facilitated regular practice sessions within the facilities and barriers to consistent practice are presented below.

### Facilitators

Four facilitating factors emerged from the data: (1) ease of using the simulators in the facility, (2) a provider is identified to establish a schedule for practice with the simulators and lead the sessions, (3) phone support, and (4) maintaining skills to be ready for an emergency.

### Simulators were acceptable

When the interviewers asked about the simulator, participants’ initial response was they had “no problem” or that the simulator was “easy” to use. Among some study participants, both MamaNatalie and NeoNatalie simulators were described as challenging to use at first; however, they reported this feeling decreased during repeated use. One challenge specific to MamaNatalie was that at least two people needed to work together to use it in a practice scenario—one to wear the model and the other to role-play the skilled birth attendant. In some smaller facilities, study participants said that shortage of staff made it difficult to bring two people together to practice.

### Someone to schedule and lead practice sessions

**Fig. 2:**
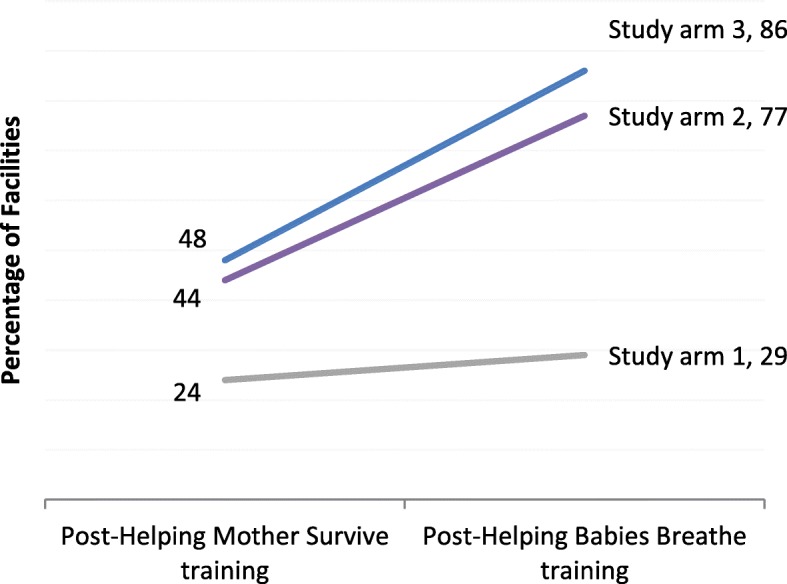
Percentage of facilities that submitted any practice documentation, by study arm and training session

**Fig. 3:**
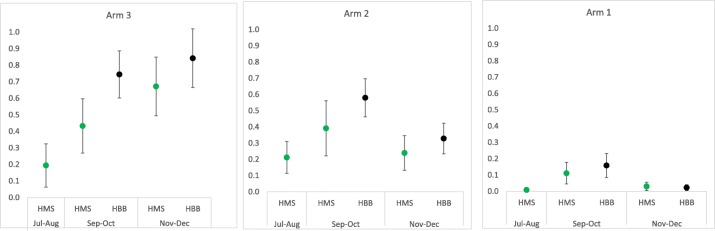
Mean number of practice sessions per month per provider and 95% confidence interval for HMS and HBB topics, by 2-month time periods, by study arm

In study arm 1, 24% of facilities submitted documentation of simulator-based practice during the period after the HMS training session, and 29% submitted documentation after the HBB training session (Fig. 2). In study arm 2, the proportion of facilities that documented any practice increased from 44% after HMS to 77% after HBB. Similarly, in study arm 3, practice documentation increased from 48% after HMS to 86% after HBB. Analyzing practice at the individual level demonstrated that the mean number of practice sessions per provider per month remained in the range of 0–1 sessions for all study arms and time points. However, after HBB when overall practice documentation increased, statistically significant differences emerged when study arms 2 and 3 are compared with study arm 1 (Fig. 3, Additional file 1).

Table 2 presents data from the provider practice logs and from the qualitative data together and presents some illustrative quotations for the themes presented here. No hospitals in study arm 1 practiced at a higher than average level; in study arm 1, at a health center that achieved higher practice, a facility manager (“in-charge”) described taking the initiative to ask staff to practice.

**Table 2:** Comparison of total mean practice session per provider and themes by level of practice achieved, facility type, and study arm

Facility type, study arm	Mean sessions per provider at the facility level (95% confidence interval)	Brief summary of themes	Illustrative quotations
Higher practice level facilities			
Hospitals, arms 2 and 3	5.5 (4.1–6.9)	*Facilitators:* Appreciation for training opportunity. Changes in clinical behavior described. Practice coordinators described as motivated and supportive. *Barriers:* Broken equipment for resuscitation, harder to realize success on NeoNatalie compared to real baby.	“We always appreciate them, we say you are doing well. …Let us continue but we need to first again, practice so that that skill does not disappear because we are getting fewer asphyxiated babies these days.”—Practice coordinator
Hospitals, arm 1 No facilities in this category			
Health centers, arms 2 and 3	8.6 (3.7–24.0)	*Facilitators:* Practice coordinators give feedback, able to describe practice in detail. Perceived reductions in intrapartum stillbirth and PPH, Desire for “skills and confidence”. Scheduling practice in advance. *Barriers:* Some colleagues more willing to practice than others, some practice coordinators are not midwives and are uncomfortable instructing midwives, scheduling conflicts, staff transfers (including practice coordinator).	“What really motivated me I was faced with it one time. …In fact the baby came out well but after some, time the condition changed but I realized it was asphyxia. …. we had to refer the child to [referral hospital] and we ended up even losing that child. …So that thing motivated me to continue practicing.”—Focus group discussion participant
Health centers, arm 1	5.8 (3.7–8.1)	*Facilitators:* In-charge motivated staff to practice. Changes in clinical behavior following training sessions. Practice during less busy times. *Barriers:* Short staffing.	“Our in charge gives us courage that… [we] are supposed to do this and this, so we have to keep on practicing.”—Focus group discussion participant
Lower practice level facilities			
Hospitals, arms 2 and 3	1.4 (0–2.7)	*Barriers:* Staffing less willing to practice. Lots of turnover, including practice coordinators. Continuing professional development requirements and grand rounds compete for practice time. Need for regular feedback to stay motivated. Providers enjoy offsite training sessions. Practice randomly, when time available, not after adverse events.	“Most of the people who were trained have gone. So when we are doing mentorship we are even like teaching. … And even those who trained, they are like less interested, they think they know.”—Practice coordinator
Hospitals, arm 1	0.1 (0–0.3)	*Barriers:* Change in clinical practice described, but skeptical of new information. Short staffing. High patient loads. Staff transfers within facility. Patients must pay for misoprostol. Simulator not realistic enough.	“We have sat with our boss and she knows what is taking place in maternity… we have complained, talked to her, had meetings, nothing has changed. So about the shortage of staff, I do not think you can do much.”—Focus group discussion participant
Health centers, arms 2 and 3	1.5 (0–3.5)	*Barriers:* Change in clinical practice following training sessions. CM not a midwife and cannot mentor midwives. Lots of staff did not complete training session. Many interruptions to practice schedule (sickness, etc.) Need more support from district trainers. Broken equipment. Staff not motivated to practice, expect compensation (“motivation”) for participation.	“They were used to being motivated. They feel as if when they sit they can get something from the training. Something, at least little either a soda.”—Practice coordinator
Health centers, arm 1	0.5 (0–2.3)	*Barriers:* Need more support and feedback. Lack of time to practice. Shortage of equipment and supplies. Stockouts.	“Monday, basically we have no clinic but patients are just many… Tuesday we have postnatal, we have antenatal, we have immunization. Wednesday we have antenatal, on Thursday we have ART clinic and outreach, on Friday, we have nutrition clinics. … So to practice, you need to find your own time outside the working hours.”—Focus group discussion participant

Just as the logs suggested that practice occurred more frequently in the facilities with a designated practice coordinator, the qualitative participants described practice coordinators as valuable for facilitating practice by giving feedback and correcting their peers when steps in a scenario needed to be done differently. In the absence of a practice coordinator, study participants suggested that the health facility in-charge could encourage practice. Although the district trainers suggested practicing at a convenient time rather than a fixed time, some participants said it was useful to pick a day of the week or a certain date in a month, commit to twice a month, or pick another time target. Study participants also recommended that instituting policies around practice and continuing education could further encourage consistent practice. Although the mobile telephone credits given to practice coordinators in study arm 3 were intended to be used for communicating with the district trainer, sometimes practice coordinators described using the airtime to call off-duty coworkers and encourage them to attend practice sessions.

### Phone support

Following the HMS training sessions, 83% of facilities in study arm 3 received at least one call from a district trainer. The mean number of calls to a health facility was 5.7 (range 0 to 17), and 57% of calls were related to practice reminders. Following the HBB training sessions, 100% of facilities received at least one call. The mean number of calls increased to 12.8 (range 1 to 46), and 58% of calls were related to practice reminders. During qualitative interviews, some practice coordinators said that the phone support motivated them to organize practice sessions. Some district trainers believed the phone calls they made to practice coordinators increased the number of times health facilities practiced and increased the likelihood that practice coordinators would complete the practice logs.

### Maintaining skills to be ready for an emergency

Practice sessions were described as necessary to maintain skills that might be needed in an emergency. Study participants expressed fear that if they did not have the skills required to deal with complications they may find themselves alone in a situation they could not handle and a patient death would result. One practice coordinator from a higher practice health center in study arm 3 motivated colleagues who were not midwives to practice scenarios by warning them, “One day a midwife will not be on duty. You will be alone, and you would not want to see a mother die in your hands.”

One suggestion study participants made to improve the program was for training and practice sessions to count towards “continuous professional development” requirements. Another suggestion was that some compensation be given for practice sessions, even as small as soda or other refreshments.

### Barriers to consistent practice

There were four main themes related to barriers to consistent practice: (1) viewing “practice” as providing routine care, (2) heavy client volume and low staffing levels, (3) lack of continued district support, and (4) the lack of compensation for practicing. Table 2 suggests that the barriers to regular practice were similar in both higher and lower practice facilities, with lower practice facilities somewhat more likely to describe a preference for the traditional, off-site training approach.

### Practice through routine clinical care

One challenge to practicing with simulators was some birth attendants’ perception that they had already mastered the competencies. For example, if they had successfully treated a client with postpartum hemorrhage or neonatal asphyxia, then continued practice might be seen as unnecessary. Others said that they had already learned these skills in school or that they had mastered the skills during the 1-day session and did not see the need to continue practicing.

### Heavy volume and low staffing

Many practice coordinators and other study participants cited “having a long queue” of clients to be seen as a barrier to practicing. One provider from the control arm said: “What I would say about practicing, I think the most convenient time to practice is when you are on duty. And when you are on duty, you are covering labor ward and nursery as one staff. So at the end of the day, you don’t even remember that you were supposed to practice.” Low staffing levels often resulted due to staff being on leave, at offsite training sessions, or temporarily transferred to another department within the facility. One district trainer said, “I trained five, three of them were transferred out...one of the two who are remaining is in maternity leave. So only one is remaining in the facility.”

### Lack of outside support

District trainers and other study participants suggested that additional supportive supervision would have been useful. Some were demotivated when they did not see their trainers again, and practice coordinators suggested that additional visits would also help build their capacity. In contrast, recognition of high-performing facilities was put forth as a potential motivator for continued practice. “One day our District Health Officer was saying that he plans to start rewarding the best performing units, the best performing health workers,” said one district trainer from study arm 2. “Probably that could be another way of motivating.”

### Lack of extra compensation

Participants commonly requested compensation or small incentives as a reward for engaging in practice sessions. Traditionally, in-service training sessions often occur off-site and come with the benefits of per diem and transport reimbursement, which were seen as a significant benefit. In this project, training sessions were conducted on-site and practice was expected to continue as part of regular schedules. Lunch compensation was provided during, but no compensation was given for, routine practice. Many practice coordinators noted that organizing practice without “motivation” (understood to be financial) was challenging.

## Discussion

In the study presented here, health facilities with practice coordinators reported higher levels of practice of simulated skills in postpartum hemorrhage care and newborn resuscitation than facilities without designated practice coordinators. This study builds on previously reported study findings which found that the training sessions and subsequent practice were feasible to implement and were associated with improvements in quality of care and patient outcomes over 9 months [21]; however, data suggested that more complex clinical skills that tend to be used infrequently, such as newborn resuscitation, might require more practice than skills that are used in everyday clinical care [21].

After the 1-day training session, maternity unit staff were asked to practice weekly. The self-reported weekly practice logs found that fewer practice sessions occurred in all three study arms. Study participants, on average, also fell short of the 2016 World Health Organization recommendation for monthly practice of intrapartum skills [2]. However, at the individual and facility level, participation varied widely.

This study found that birth attendants valued on-site simulation-based practice and described various factors that could explain the higher or lower levels of practice achieved. Workload, staff turnover, intrinsic and extrinsic motivation, and supervision affected willingness and motivation to practice with a simulator. While short staffing and heavy workloads were common across all facilities, some differences emerged by study arm and when comparing the lower- and higher-level facilities. Having a practice coordinator in the facility to encourage practice sessions was helpful, although some study participants questioned their role and the incentive system. Although altruistic intentions, such as a desire to improve patient care and be prepared for emergencies, can be motivating, it is also important to consider extrinsic motivations, such as food, monetary benefits, recognition, and continuing education credits.

One study limitation is that the practice logs were self-reported and may have either underestimated true levels of practice, if practice coordinators forgot to record practice sessions, or overestimated true levels of practice, if they recorded more frequent sessions than were actually conducted, because of social desirability bias. Another limitation is that although efforts were made to schedule interviews and focus group discussions in advance, this was not always possible and provider participation was often limited to those who were available when interviewers arrived; the study team did not formally track refusal rates. Therefore, the study participants included in focus group discussions in the “higher practice” facilities were not necessarily those who had participated in practice sessions. This also meant that some study participants were distracted by their workload and would step away from the discussion at times. Sometimes practice coordinators were unavailable for interview; as shown in Table 1, we intended to interview 12 from higher practice facilities but were able to interview only 6. In a few cases, practice coordinators participated in focus group discussions, rather than individual interviews, which could influence other study participants’ feedback on the program, practice, and the support of the practice coordinator. Member checking was not deemed feasible in this study.

This study fills a practical need to better understand how to implement and sustain capacity-building programs within health systems in low- and middle-income countries, moving beyond clinical trials, which have demonstrated the efficacy of these interventions under controlled conditions [20]. One strength of this study is that we collected data from several types of people involved in the implementation of the intervention and from multiple qualitative and quantitative data sources. We also obtained perspectives from those involved in implementing the program and the program participants. To minimize socially desirable responses in the interviews and focus group discussions, the qualitative data collection team was separate from the study implementation team.

The research literature around training approaches for obstetric emergencies is limited [27], and the body of published research around simulator-based practice in sub-Saharan Africa and similar settings is even more so [28]. A literature search yielded no articles that presented practice frequency in the manner that we have, although some alluded to the frequency of practice sessions. Another challenge to comparing this study to previous studies is that the scale of the implementation of many published studies was small, while this Uganda study included 125 facilities in 12 districts, some in remote areas of two larger regions, and approximately 800 health care workers. Some qualitative studies show similar findings. In a study of HBB scale up in Tanzania, health care providers gave positive feedback on the training approach, but it was challenging to achieve consistent practice within health facilities, and some providers requested more compensation for participating in the program [29]. In a study in year 2000 in one of the districts in which this study was conducted, Kaye found that poor quality of care was attributed to absence of hands-on in-service training sessions, combined with understaffing and lack of clinical management guidelines [30] . More recently, a survey of Uganda health workers highlighted concerns about staff absenteeism, staffing levels at rural facilities, and quality of services [31].

In a systematic review of factors related to the “third delay” in maternal health care (the delay of receiving services after arriving at the health facility), Knight found that issues related to staff knowledge and skills was the most common factor, such as not being up-to-date on management of emergencies or not recognizing emergencies promptly enough [32]. Other factors that were mentioned echoed those described by the participants in this study as barriers to consistent practice sessions: short staffing and low motivation due to feeling overwhelmed at work. Knight found lack of equipment to be a barrier. The Uganda study supplied the necessary equipment to all facilitates so that this would not be a barrier to provider practice. Evaluating health care providers’ experiences with an HBB program in Tanzania through qualitative methods, Isangula et al. also found that they valued the training content and they identified several factors that limited the program’s impact, including internal staff transfers and rotations, broken equipment, variability in the levels of simulation-based practice completed, and complaints about the lack of per diem compensation during on-site training sessions [33].

Some study participants suggested providers would respond favorably to incentives to practice—even something as small as refreshments—or recognition for facilities that achieved higher practice. Research around incentives, both financial and non-financial, has yielded mixed results; reviews of conditional cash transfers and similar programs for health care consumers have suggested that individuals can be responsive to small, immediate rewards [34]. The benefit of recognition ceremonies and awards (such as certificates and plaques) to motivate provider behavior is consistent with Jhpiego’s experience implementing quality improvement initiatives through the standards-based management and recognition (SBM-R) approach in 30 countries [35, 36]. After this project ended, the study team organized a dissemination event in each district to present study results to participants and stakeholders, as well as provide recognition in the form of certificates or engraved plaques for high-performing districts, health facilities, district trainers, practice coordinators, and proxies.

Opportunities for continuing professional development are thought to improve health worker motivation and morale [37]. The study identified opportunities for increasing motivation through integration with continuing professional development (CPD) programs within the Ugandan health system. Currently, health professionals in Uganda are required to complete 50 h of accredited CPD every year when seeking re-licensure [38]. Study participants said that practice could be increased if participation in LDHF allowed them to receive CPD credits. In order to pursue this further, there is a need to strongly engage and advocate with leading regulatory bodies in the country to facilitate support.

## Conclusions

In Uganda, birth attendants and other maternity unit staff valued the opportunity to use simulation-based practice to maintain life-saving skills. Workload, staff turnover, intrinsic and extrinsic motivation, and supervision affected study participants’ willingness and motivation to practice with a simulator. Designating a person in the facility to lead regular practice sessions and providing support to that person contributed to increased frequency of practice. Health centers reported higher frequency of average practice sessions per health care provider, suggesting that future work should investigate how to increase participation in simulation-based practice sessions at hospitals. This also suggests that the LDHF intervention may be most suitable or acceptable for providers at health centers. Health systems should explore how to integrate the LDHF approach into existing continuing professional development activities.

## Additional file

Additional file 1: **Table S1.** Mean number of reported practice sessions per provider for HMS and HBB topics and 95% confidence interval (CI), by study arm and two-month intervals. (DOCX 16 kb)

## Abbreviations

HBB: Helping Babies Breathe
HMS BAB: Helping Mothers Survive Bleeding after Birth
LDHF: Low-dose high-frequency
WHO: World Health Organization

## Footnotes

1 The training curriculum is available online: https://hms.jhpiego.org/

2 No HBB topics were practiced during July–August because this was prior to the HBB training sessions
