# Supplementary material for: “Practice so that the skill does not disappear”: mixed methods evaluation of simulator-based learning for midwives in Uganda
Source: Hum Resour Health. 2019 Mar 29;17:24. doi: 10.1186/s12960-019-0350-z (PMC6440002; doi:10.1186/s12960-019-0350-z)
Supplement: Supplementary file 1 — Table S1. Mean number of reported practice sessions per provider for HMS and HBB topics and 95% confidence interval (CI), by study arm and two-month intervals. (DOCX 16 kb) [file 12960_2019_350_MOESM1_ESM.docx]

Table S1 Mean number of reported practice sessions per provider for HMS and HBB topics and 95% confidence interval (CI), by study arm and two-month intervals

| **Study arm & Topic** | **July-August** | **September-October** | **November-December** |
| --- | --- | --- | --- |
| HMS |  |  |  |
| Study arm 1 | 0.01 (95% CI 0.00-0.00) | 0.11 (95% CI 0.04-0.18) | 0.03 (95% CI 0.00-0.05) |
| Study arm 2 | 0.21 (95% CI 0.11-0.31) | 0.39 (95% CI 0.22-0.56) | 0.24 (95% CI 0.13-0.35) |
| Study arm 3 | 0.19 (95% CI 0.06-0.32) | 0.43 (95% CI 0.27-0.60 | 0.67 (95% CI 0.49-0.85) |
| HBB |  |  |  |
| Study arm 1 | n/a | 0.16 (95% CI 0.08-0.23) | 0.02 (95% CI 0.00-0004) |
| Study arm 2 | n/a | 0.58 (95% CI 0.46-0.70) | 0.33 (95% CI 0.23-0.42) |
| Study arm 3 | n/a | 0.74 (95% CI 0.60-0.89) | 0.84 (95% CI 0.66-1.02) |

Notes: (a) HMS BAB, Helping Mothers Survive Bleeding After Birth. HBB, Helping Babies Breathe. 95% CIs are shown in parentheses.
